# Non-Injurious Neonatal Hypoxia Confers Resistance to Brain Senescence in Aged Male Rats

**DOI:** 10.1371/journal.pone.0048828

**Published:** 2012-11-16

**Authors:** Nicolas Martin, Carine Bossenmeyer-Pourié, Violette Koziel, Rozat Jazi, Sandra Audonnet, Paul Vert, Jean-Louis Guéant, Jean-Luc Daval, Grégory Pourié

**Affiliations:** 1 Inserm U954, Vandoeuvre-lès-Nancy, France; 2 Université de Lorraine, Faculté de Médecine, Vandoeuvre-lès-Nancy, France; 3 Service de Médecine Néonatale, Maternité Régionale Universitaire, Nancy, France; 4 IRCCS, Oasi Maria S.S., Institute for Research on Mental Retardation and Brain Aging, Troina (EN), Italy; University of South Florida, United States of America

## Abstract

Whereas brief acute or intermittent episodes of hypoxia have been shown to exert a protective role in the central nervous system and to stimulate neurogenesis, other studies suggest that early hypoxia may constitute a risk factor that influences the future development of mental disorders. We therefore investigated the effects of a neonatal “conditioning-like” hypoxia (100% N_2_, 5 min) on the brain and the cognitive outcomes of rats until 720 days of age (physiologic senescence). We confirmed that such a short hypoxia led to brain neurogenesis within the ensuing weeks, along with reduced apoptosis in the hippocampus involving activation of Erk1/2 and repression of p38 and death-associated protein (DAP) kinase. At 21 days of age, increased thicknesses and cell densities were recorded in various subregions, with strong synapsin activation. During aging, previous exposure to neonatal hypoxia was associated with enhanced memory retrieval scores specifically in males, better preservation of their brain integrity than controls, reduced age-related apoptosis, larger hippocampal cell layers, and higher expression of glutamatergic and GABAergic markers. These changes were accompanied with a marked expression of synapsin proteins, mainly of their phosphorylated active forms which constitute major players of synapse function and plasticity, and with increases of their key regulators, i.e. Erk1/2, the transcription factor EGR-1/Zif-268 and Src kinase. Moreover, the significantly higher interactions between PSD-95 scaffolding protein and NMDA receptors measured in the hippocampus of 720-day-old male animals strengthen the conclusion of increased synaptic functional activity and plasticity associated with neonatal hypoxia. Thus, early non-injurious hypoxia may trigger beneficial long term effects conferring higher resistance to senescence in aged male rats, with a better preservation of cognitive functions.

## Introduction

Adverse environmental conditions during early development have been shown to influence health throughout life [1]–[3]. Effects of the early cues on ensuing disease risk are not limited to the intrauterine conditions, since the postnatal environment may also influence health outcome and subsequent brain functioning. For example, adverse events during early childhood were found to be a strong predictor of multiple risk factors for various chronic diseases later in life [3]. Conversely, it is documented that postnatal exposure to an enriched environment has beneficial consequences [4]. Neuroadaptations and plasticity may include structural reactions (e.g., neurogenesis or cell death) and/or functional modifications in sensitive brain regions (e.g., long-term potentiation (LTP), long-term depression (LTD), reorganization of synaptic protein dynamics etc.). In this respect, exposure to transient oxygen deprivation frequently occurs around birth. If sustained neonatal hypoxia/ischemia remains a major cause of brain injury and neurological disabilities, brief acute or intermittent episodes of hypoxia have been shown to exert a protective role in the central nervous system. Indeed, hypoxic conditioning can prevent the deleterious impact of a subsequent, more severe stimulus, by inducing tolerance [5], [6]. Among the mechanisms involved, it was shown that mild/brief hypoxia triggers the generation of new neurons issued from germinative areas, such as the dentate gyrus in the hippocampus and the subventricular zone [7]–[9], corroborating other studies in adult animals where neurogenesis occurs not only as a transient repair mechanism but appears to be a continuous phenomenon over lifespan [10], [11]. Interestingly, conditioning-like brief postnatal hypoxia was associated in previous studies with improved functional scores in young rats, along with increased size of various hippocampal regions, suggesting the presence of surnumerary neurons in some brain structures [9], [12]. However, several studies have linked neonatal hypoxia with increased risk of psychotic disorders, such as schizophrenia, later in life [13], [14]. The changes triggered in the brain stimulated by “pathologic” neurogenesis may lead to abnormal communications in exquisitely regulated neural networks. This could in turn cause mental diseases, altered cognitive functions or premature brain aging [15]. In this respect, a particular attention should be paid to the relative distribution of the different neuronal phenotypes that constitute brain circuits and govern the balance between excitatory and inhibitory connections [16]–[19].

We therefore monitored brain functional outcome and tissular effects of a brief neonatal hypoxia – that has been demonstrated to trigger neurogenesis – in rats until the age of 2 years, which corresponds to senescence in normal conditions. Because it has been documented that males and females are affected differently by hypoxia and related pathologic conditions [12], [20], [21], the two genders were investigated separately.

## Results

### Short-term Effects of Transient Neonatal Hypoxia

In these experiments, since no significant differences were found between males and females (ANOVA), results were retrospectively pooled across sex.

#### Blood gases

In our experimental conditions, acute hypoxia for 5 min strongly reduced PO2 (40.6±5.3 *vs* 62.5±6.1 mm Hg, means ± s.d., n = 5, *P*<0.01) at the end of exposure, while it increased PCO2 (49.2±6.1 *vs* 33.7±5.8 mm Hg, *P*<0.05) and pH (7.23±0.04 *vs* 7.38±0.02, *P*<0.01).

#### Key proteins involved in cell death and survival

**Figure 1:**
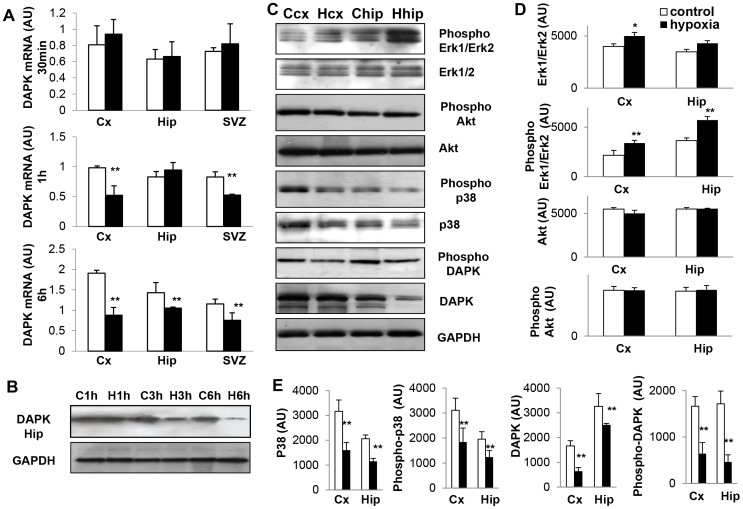
Short-term changes in major signaling proteins in response to transient hypoxia. (**A**) Temporal profiles of DAPK mRNAs in frontal cortex (Cx), hippocampus (Hip) and subventricular zone (SVZ) following hypoxia and in matched controls. Data are reported as means ± SD (n = 3). Statistically significant differences between hypoxia and controls: **P<0.01. (**B**) Temporal profiles of total DAPK expression in the hippocampus at 1, 3 and 6 hours following hypoxia (H) and matched controls (C). Experiments were performed three times with similar results. (**C**) Expression profiles of total and phosphorylated Erk1/2 (p44 and p42) MAP kinases, Akt and phospho-Akt, p38 and phospho-p38, DAPK and phospho-DAPK, 3 hours after exposure to hypoxia (H) and in matched controls (C) in frontal cortex (cx) and hippocampus (hip). Experiments were performed five times with similar results. (**D,E**) Corresponding densitometric analyses of protein expression in arbitrary units (AU). Data are reported in arbitrary units (AU) as means ± SD (n = 5). Statistically significant differences between hypoxia and controls: *P<0.05; **P<0.01.

Transcript amounts of the developmental death-associated protein (DAP) kinase progressively decreased between 30 min and 6 h after exposure to hypoxia in the brain structures investigated, suggesting a protective mechanism (Fig. 1A). This observation was confirmed at the protein level by Western blotting (Fig. 1B), with a reduced expression of the active form, i.e. phospho-DAP kinase (Fig. 1C,E).

Three hours after hypoxia, total amounts of Erk1/2 MAP kinases were not significantly affected, but the expression of phopho-Erk1/2 was significantly increased in response to hypoxia. The reverse was observed for phospho-p38, whereas phospho-Akt expression did not vary (Fig. 1C,D).

### Medium-term Effects of Transient Neonatal Hypoxia

#### Cell proliferation

**Figure 2:**
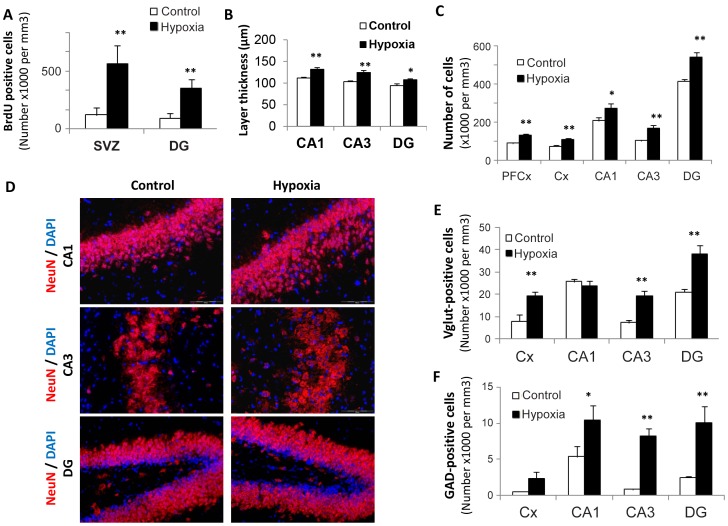
Medium-term changes in 21-day-old rat brains after transient neonatal hypoxia. (**A**) Number of BrdU-positive cells recorded in the subventricular zone and in the dentate gyrus of control rats and those exposed to brief hypoxia after birth. Results are expressed as cell numbers per mm^3^ (n = 5). Statistically significant difference between hypoxia and controls: **P<0.01. (**B,C**) Layer thicknesses (µm) and cell numbers per mm^3^ in various brain structures (CA1, CA3 = hippocampus Hammon’s horn layers; DG = dentate gyrus, Cx = frontal cortex, PFCx = prefrontal cortex), n = 5, *P<0.05, **P<0.01. (**D**) Immunohistochemical staining of NeuN, a marker of mature neurons, in various hippocampus layers showing hypoxia-associated increases in thickness and/or cell densities. Cells were counterstained by Dapi (x20 magnification). (**E,F**) Numbers of glutamatergic (VGluT2-positive) cells and of GABAergic (GAD65-positve) cells in various brain structures, n = 5, *P<0.05, **P<0.01.

The number of BrdU-positive cells within the subventricular zone (SVZ) and the subgranular layer of the dentate gyrus (DG) was found to be respectively 3.5 and 2.4 fold increased in rats previously exposed to hypoxia as compared to controls, with the strongest BrdU incorporation recorded by three weeks post-exposure in both structures (Figure 2A, n = 5 per group, *P*<0.001). Although not investigated in the present study, previous experiments showed that hypoxia-associated cell proliferation gave rise to functional neurons [9]. Concomitantly, thicknesses of hippocampal CA1, CA3 layers and dentate gyrus were significantly augmented (ranging from 15 to 20%) in 21-day-old hypoxic rats compared to controls (Fig. 2B,D), whereas cell density was found to be higher (20–35%) in all structures examined (Fig. 2C,D). As illustrated in Fig. 2D, cell labeling with NeuN suggests that the surnumerary cells in hypoxic rats are mature neurons. Finally, both glutamatergic and GABAergic phenotypes appeared to be increased in most structures following hypoxia, as shown by the number of cells expressing either the vesicular glutamate transporter 2 (VGluT2) or the glutamate decarboxylase 65 (GAD65) GABAergic synaptic isoform (Fig. 2E,F).

#### Functional synaptic markers

**Figure 3:**
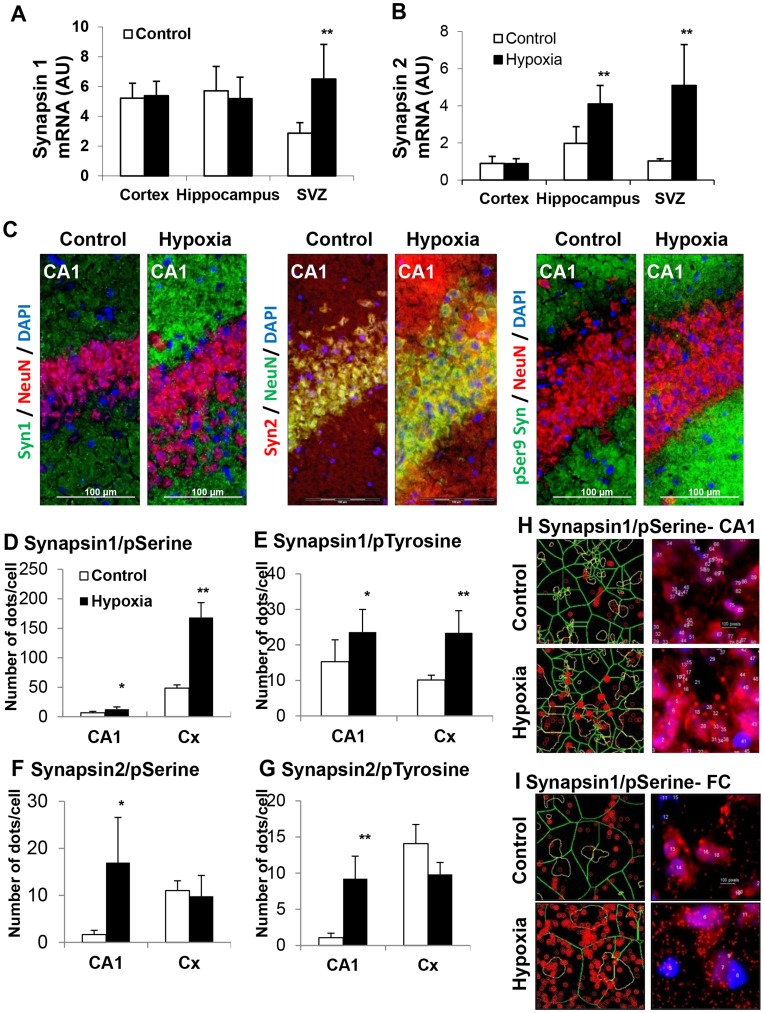
Changes in functional synaptic markers, synapsins I and II, in response to hypoxia. (**A,B**) Synapsins I and II mRNA levels in various brain structures of rats 30 min after exposure to hypoxia and their matched controls (n = 3, **P<0.01). (**C**) Immunohistological detection of synapsin I, synapsin II and phospho-synapsins (Ser^9^) in the CA1 layer of 21-day-old rats previously exposed to hypoxia and their matched controls (cells were counterstained by Dapi (blue) and by the neuronal protein NeuN (red). (**D–G**) *In situ* coupling between synapsins with phospho-Ser^9^ and phospho-Tyr^301^, as monitored by the Duolink® “proximity ligation” assay: number of dots per cell in CA1 and frontal cortex (Cx) of 21-day-old control and hypoxic rats. Experiments were performed in triplicate (*P<0.05, **P<0.01). (**H,I**) Illustration of *in situ* association between synapsin I and phospho-Ser^9^ in the CA1 layer and the frontal cortex (Cx) of control and hypoxic rats, as revealed by red fluorescent dots in the Duolink® “proximity ligation” assay. Experiments were performed in triplicate, leading to similar results.

Transcripts of neuronal plasticity proteins synapsins were significantly elevated as early as 30 min after exposure to hypoxia in the SVZ for synapsin I (2-fold increase) and in hippocampus and SVZ for synapsin II (2- and 5-fold increase, respectively) (Fig. 3A,B). At 21 days of age, synapsin I, synapsin II and phospho-synapsins (Ser^9^) were more elevated in the hippocampus of rats previously exposed to hypoxia, as illustrated by immunochemistry in the CA1 layer (Fig. 3C). In addition, the “proximity ligation” assay (Duolink®) confirmed hypoxia-associated increased expression of phospho-synapsins (Ser^9^ and Tyr^301^) in both CA1 layer and frontal cortex for synapsin I, and in the CA1 layer for synapsin II (Fig. 3D–I).

### Long-term Effects of Transient Neonatal Hypoxia during Aging

#### Locomotor capacities, learning, memory, and metabolic activities

**Figure 4:**
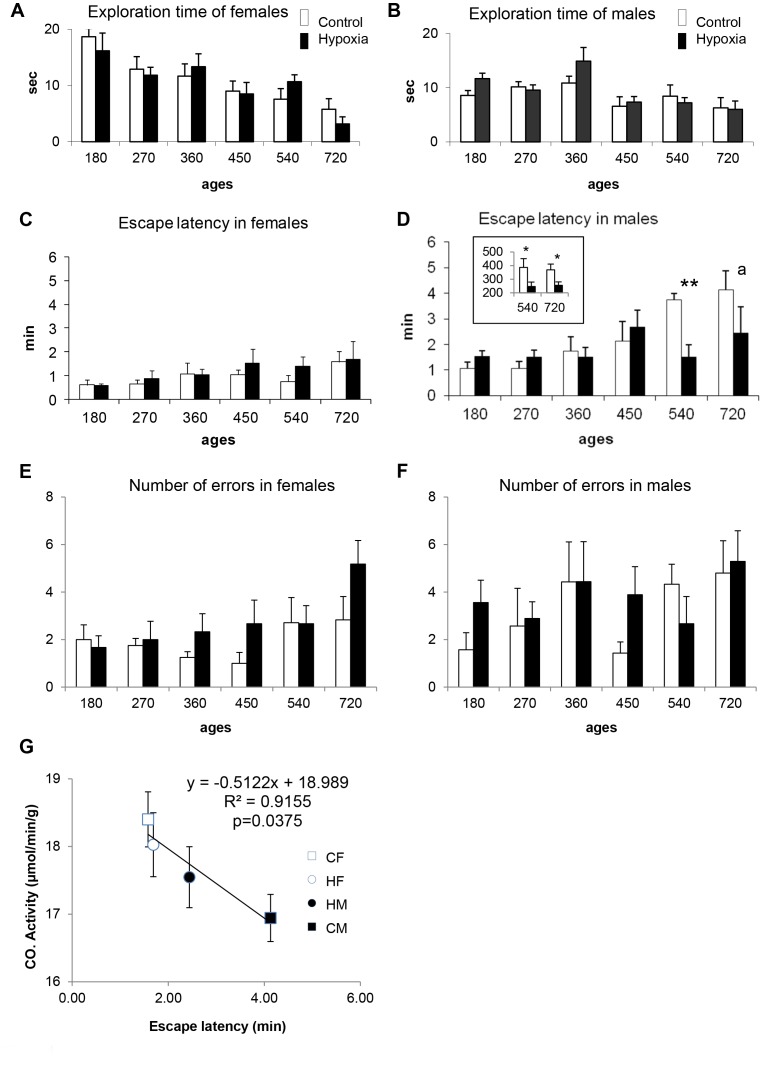
Effects of neonatal hypoxia on behavioral capacities between 180 and 720 days of age. (**A–B**) Analysis of spontaneous locomotor activity in the open-field, with total exploration times for females (A) and males (B). (**C–F**) Memory retrieval scores in the multiple-T maze, as shown by escape latency and number of errors, with females’ scores (C,E) and males’ scores (D,F). Data are reported as means ± s.d. and were obtained from 15 individuals per experimental group, except at 720days. **P = 0.0001; ^a^ *P* = 0.08 (reveals only a tendency due to an elevated mortality at this age (one-way ANOVA). The inlet in panel D shows the total distance moved (in centimeters) by males in the maze at 540 and 720 days of age, with significant differences *P = 0.033 and 0.035, respectively (**G**) Significant correlation obtained at 720 days between cytochrome oxidase (CO) activity in the dentate gyrus and the functional scores in the multiple T-maze reflected by escape latencies for males (black), females (white), previously exposed (circles) or not (squares) to hypoxia. Note the position of hypoxic males’ values detached from control males’ values and reaching overall females’ values.

Regarding spontaneous activity in the open-field between 180 and 720 days of age, no statistically significant differences were found between the two experimental groups (i.e., controls and hypoxia), either in the exploration time (Fig. 4A,B) or in the number of rearings (not shown). Taken together, these data showed that rats were not affected in their locomotion.

When rat pups were placed for 5 consecutive days (between 14 and 18 days of age) in a multiple T-maze for learning a visuo-spatial task, all of them were successful, whatever the experimental group concerned, in good accordance with previous report [12]. The time spent to achieve the goal as well as the number of errors made gradually decreased over time similarly in control and hypoxia groups. In addition, male and female rats performed equally in this test (not shown).

Memory retrieval, involving the participation of hippocampus and prefrontal/frontal cortices, was then assessed in the same multiple T-maze between 180 and 720 days of age. As a whole, scores tended to be better in control females than in control males, a phenomenon that reached statistical significance starting from 450 days, when males began to need longer time to escape the maze (Fig. 4C,D). Whereas no significant difference in escape latency emerged at any time between control and hypoxic females, males previously exposed to transient neonatal hypoxia displayed significantly higher scores than controls at 540 days (*P* = 0.0001). Scores tended to be better at 720 days, but statistical significance could not be reached (*P* = 0.08), probably because of age-related mortality (3 among 7 in controls and 2 among 9 in hypoxia group) that reduced the number of animals for statistical analysis. The number of errors varied slightly according to the ages of the animals at the time of the test, with no significant differences between control and hypoxia groups (Fig. 4E,F). In addition, the total distance moved in the maze at 540 and 720 days of age by control males was significantly longer than the distance moved by hypoxic males. Similar measurements performed at other ages in males and in females did not reveal any significant difference (ANOVA summary: F(1,13) = 0.005 to 3.072, *P* = 0.103). Because they are strongly associated, the various parameters must be considered all together. Indeed, a lower escape latency, a shorter distance moved, along with no difference in errors committed by hypoxic males compared with their matched controls indicate that hypoxic rats had less hesitation (body rotation in relation with distance moved) and more direct decision making at cross points than controls. Such behaviors would reflect better memory retrieval performances (memory scores) for hypoxic males.

Because brain adaptation in response to various environmental conditions may be related, at least partly, to metabolic changes, overall metabolic activity was regionally assessed by the measurement of cytochrome oxidase activity at 720 days of age. As shown in Table 1, no significant differences emerged between control and hypoxia groups, whatever the brain structure examined. Nevertheless, ANOVA revealed a sex effect in the CA3 layer of the hippocampus and the dentate gyrus, with higher activities in females (*P*<0.05). Interestingly, a significant correlation (*P* = 0.037) was found between cytochrome oxidase activity in the dentate gyrus − a critical structure for memory retrieval − and functional scores reached by the four experimental groups, i.e., males and females, subjected or not subjected to neonatal hypoxia (Fig. 4G).

**Table 1:** Cytochrome oxidase activity in cognitive brain structures at 720 days of age.

	Females		Males	
	Controls	Hypoxia	Controls	Hypoxia
CA1 layer	16.90±1.11	17.40±0.38	16.85±0.45	16.95±0.72
CA3 layer*	16.90±0.80	16.65±0.62	15.60±0.70	15.82±0.61
Dentate gyrus*	18.41±0.41	18.00±0.47	16.94±0.35	17.55±0.45
Frontal cortex	16.75±0.55	16.90±0.52	15.92±0.49	16.22±0.63
Prefrontal cortex	17.50±0.51	16.88±0.70	16.82±0.90	15.80±0.52

Cytochrome oxidase activity – reflecting overall metabolic activity – was quantified by histochemistry on sagittal brain sections. Data are expressed as µmol/min per g of tissue (means ± SEM) and were obtained from 3 to 4 individuals in each group.

* Significant sex effect, *P*<0.05 (ANOVA).

#### Histological correlates

**Figure 5:**
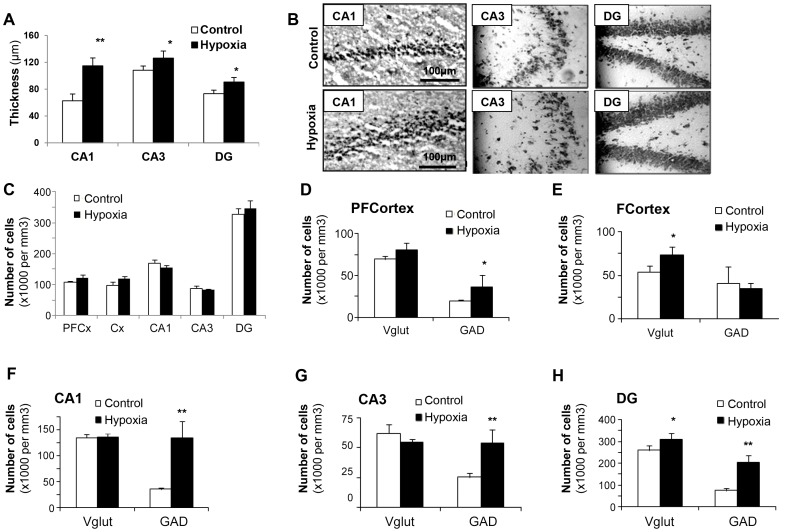
Effects of neonatal hypoxia on brain histological characteristics in 720-day-old male rats. (**A–C**) Layer thicknesses (µm), illustrations in the hippocampus layers after tissue coloration by thionine, and cell numbers per mm^3^ in various brain structures, n = 5, *P<0.05, **P<0.01. (**D–H**) Numbers of glutamatergic (VGluT2-positive) cells and of GABAergic (GAD65-positve) cells in various brain areas of control and hypoxic male rats (PFCortex = prefrontal cortex, FCortex = frontal cortex, DG = dentate gyrus), n = 5, *P<0.05, **P<0.01.

In good agreement with behavioral observations, transient neonatal hypoxia did not affect major brain histological characteristics of females at 720 days of age, as illustrated in Figure S1. By contrast, increased layer thickness could be depicted in CA1, CA3 and dentate gyrus in the brains of 720-day-old male rats previously exposed to hypoxia, with 90, 10 and 15% increase, respectively (Fig. 5A,B), but with no changes in cell densities in the various brain areas examined (Fig. 5C). In addition, increases of glutamatergic and/or GABAergic markers could be observed in most areas involved in hippocampus-dependent learning and memory processes (Fig. 5D–H). Ki67 immunochemistry revealed the persistent presence of proliferating cells in the two major germinative brain areas, i.e. the dentate gyrus and the SVZ, of aged rats, with no significant differences between control and hypoxic rat groups: 34 993±1 225 *versus* 40 406±4 415 positive cells per mm^3^ in the dentate gyrus (n = 4, *P* = 0.291, F(1.6) = 1.34) and 22 906±2 418 *versus* 16 862±2 687 positive cells per mm^3^ in the SVZ (n = 4, *P* = 0.145, F(1.6) = 2.79) (see Figure S2 for illustration).

**Figure 6:**
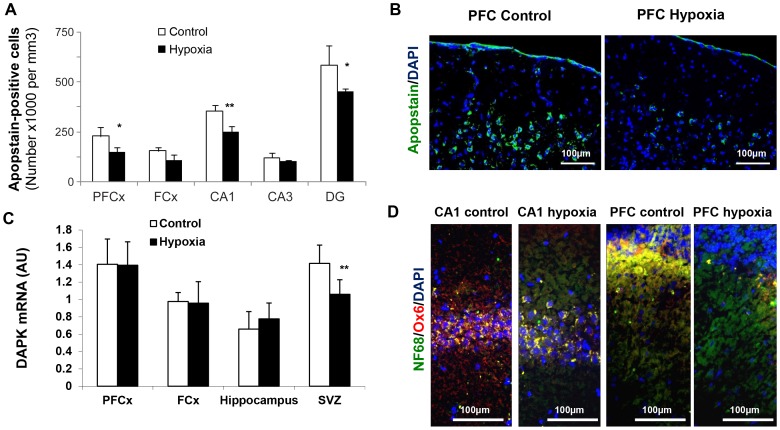
Decreased brain apoptosis and neuroinflammation in 720-day-old male rats previously exposed to neonatal hypoxia. (**A**) Apoptotic cells in various key brain areas analyzed by means of the Apostain F7-26 specific monoclonal antibody against single-stranded DNA. Data are expressed as number of positive cells per mm^3^ (means ± s.d.) and were obtained from 3 individuals in each group. Statistically significant differences between control and hypoxia groups: *P<0.05, **P<0.01. (**B**) Illustration in the prefrontal (PF) cortex. (**C**) DAP kinase mRNA levels in various brain structures, with a significant decrease in the neurogenic subventricular zone (**P<0.01). (**D**) Noticeable hypoxia-associated decreased neuroinflammation in the CA1 layer and in the prefrontal cortex in male rats belonging to the hypoxia group, as shown by the use of Ox-6 antibody (cells were counterstained by Dapi and the neuronal marker NF68).

Concomitantly, apoptosis and inflammation, as depicted by Apostain and Ox-6, respectively, were lower in aged males previously subjected to hypoxia (Fig. 6A,B,D), whereas transcription of the apoptosis-promoting DAP kinase was significantly reduced in the SVZ, where neurogenesis is known to persist over lifespan (Fig. 6C).

#### Synaptic functions and plasticity

**Figure 7:**
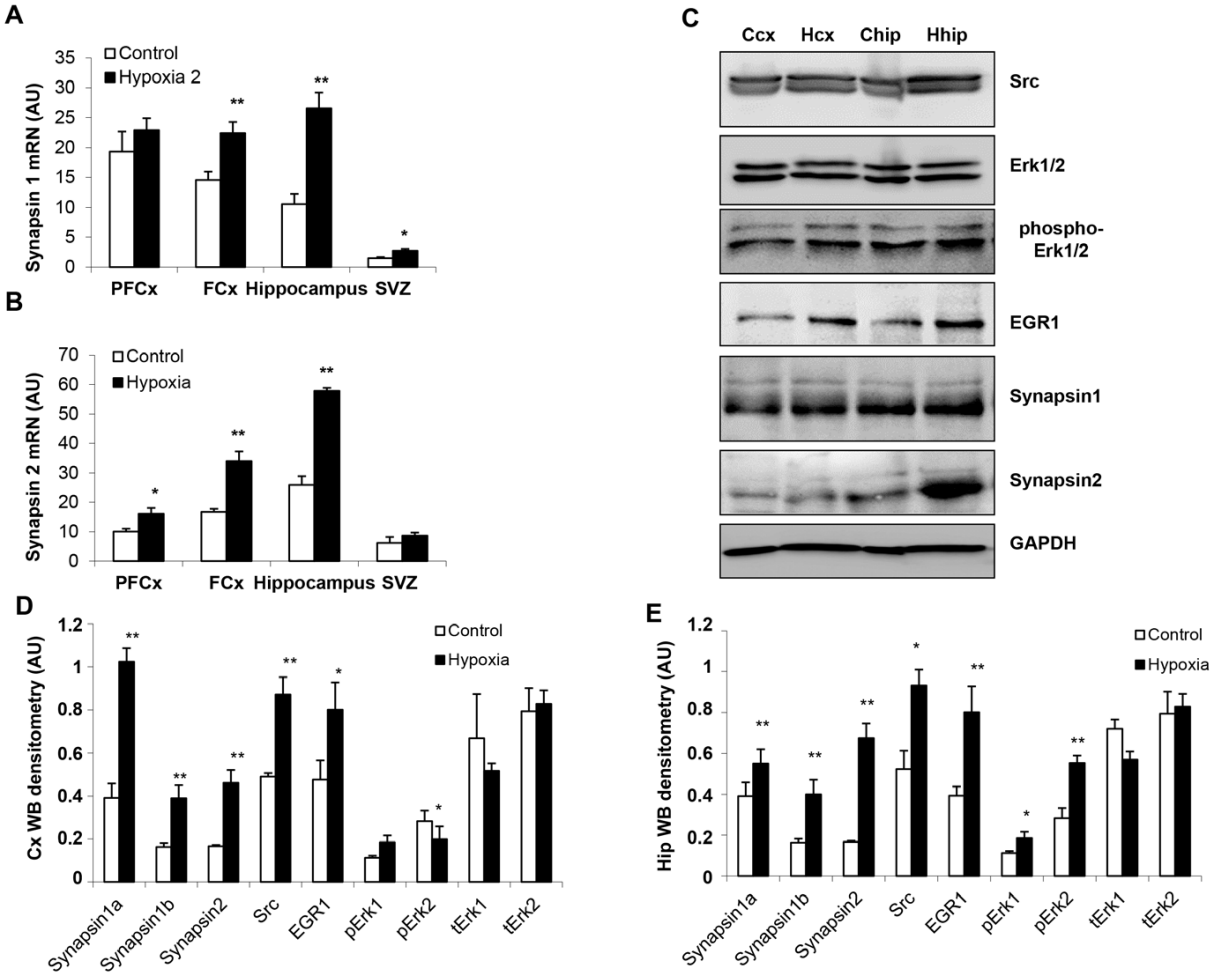
Effects of neonatal hypoxia on synapsin-related synaptic function and plasticity in 720-day-old male rats. (**A,B**) Synapsins I and II mRNA levels in various brain structures (PFCx = prefrontal cortex, FCx = frontal cortex, SVZ = subventricular zone) in control and hypoxia groups (n = 3, *P<0.05, **P<0.01). (**C**) Western blot analysis of synapsins and their regulatory proteins, in frontal cortex (cx) and hippocampus (hip) of control (C) and hypoxic (H) rats. (**D,E**) Densitometric analysis of western blots presented in panel C. Data are expressed in arbitrary units (AU) as means ± SD (n = 3, *P<0.05, **P<0.01).

**Figure 8:**
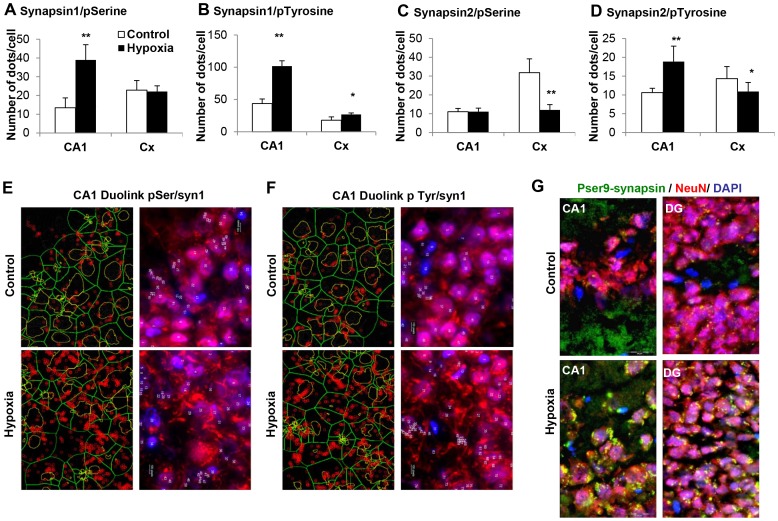
Effects of neonatal hypoxia on synapsin phosphorylation in 720-day-old male rats. (**A–D**) *In situ* coupling between synapsins with phospho-Ser^9^ and phospho-Tyr^301^, as monitored by the Duolink® “proximity ligation” assay: number of dots per cell in CA1 and frontal cortex (Cx) of control and hypoxic male rats. Experiments were performed in triplicate (*P<0.05, **P<0.01). (**E–G**) Illustrations of *in situ* association between synapsin I and phospho-Ser^9^ and phospho-Tyr^301^ in the CA1 layer and the dentate gyrus (DG, panel G) of control and hypoxic rats. Cells were counterstained with Dapi and NeuN.

In parallel to improved cognition and persisting brain histological changes associated with neonatal hypoxia in male rats, mRNA amounts and protein expression of synapsins were found to be increased in various brain structures, especially in the hippocampus (Fig. 7A–E). Moreover, as illustrated by histochemistry and documented by Duolink®, synapsin phosphorylation differed between control and hypoxia groups (Fig. 8). Augmented Tyr^301^ phosphorylation of synapsin I was evidenced in the CA1 layer and frontal cortex, whereas higher Ser^9^ phosphorylation was observed only in CA1 (Fig. 8A,B,E,F). Regarding synapsin II, only Tyr^301^ phosphorylation was significantly augmented in CA1, while its phosphorylation at both residues (i.e., Ser^9^ and Tyr^301^) was reduced in the frontal cortex (Fig. 8C,D).

The Erk-dependent serine phosphorylation of synapsins plays a key role in the modulation of their functionality [22]. It contributes to the cellular distribution of synapsins, the establishment of functional synaptic connections, the cycling of synaptic vesicles and the release of neurotransmitters [23], [24]. Furthermore, synapsins are downstream targets for the zinc finger transcription factor EGR-1/Zif-268 that regulates the expression of their genes [25], [26], and it was reported that Erk1/2 pathway stimulates expression and biological activity of the transcriptional regulator EGR-1 [27]. Finally, it has been shown that Tyr^301^ phosphorylation of synapsin I by Src kinase regulates synaptic-vesicle trafficking [28]. We therefore additionally assessed the expression of these various proteins in the brain of 720-day-old male rats. In good agreement, Src, phospho-Erk (mainly phopho-p42), and EGR-1 were found to be increased in the hypoxia group of aged male rats, especially in the key structure corresponding to the hippocampus (Fig. 7C–E). These observations may explain the downstream increased expression and phosphorylation of synapsins. The corresponding proteins remained unaffected in females at the same age (Figure S1D).

**Figure 9:**
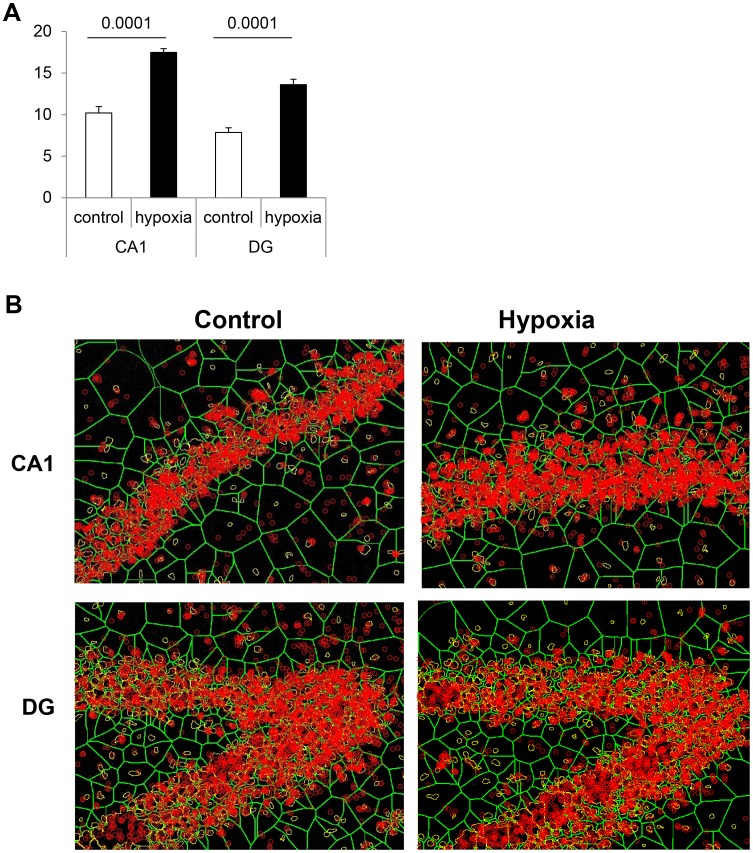
Effects of neonatal hypoxia on the *in situ* interaction between NR2A and PSD-95 in the hippocampus of 720-day-old male rats. (A) Quantification of the association between NR2A and PSD-95, as monitored by the Duolink® “proximity ligation” assay: number of dots per cell in CA1 and dentate gyrus (DG) of control and hypoxic male rats (N = 6/group, with a minimum of 230 cells analyzed/group). Note that the interaction in hypoxia group is 1.72 fold increased. (B) Illustrations of *in situ* association between NR2A and PSD-95 in the CA1 layer and the dentate gyrus (DG) of control and hypoxic rats.

At neuronal excitatory synapses, NMDA receptors are trafficked and clustered by the post-synaptic density (PSD)-95 membrane associated scaffolding protein that has multifaceted functions in regulating synaptic transmission and synaptic plasticity [29], [30]. Since it has been shown that PSD-95 binds directly to NR2 (preferentially NR2A) receptor subunits, we used *in situ* proximity ligation assay to evaluate the interaction between these two components in the hippocampus of aged male rats, as an additional index of functional activity and plasticity. As shown in Fig. 9, the interaction between PSD-95 and NR2A subunits was significantly enhanced (1.72 fold) in male rats previously subjected to neonatal hypoxia as compared to their matched controls.

## Discussion

It is now well established that neurogenesis occurs throughout the life in mammals, and that neural stem cells reside in the adult central nervous system [31]. Moreover, it has been documented that brain injuries, depending on their characteristics, can trigger neurogenesis [32]. In recent studies, we showed that a short (5 min) neonatal hypoxia in rats can activate endogenous brain neurogenesis that secondarily gives rise to a significant functional gain [9], [12]. In good agreement, Lu et al. [33] reported that postnatal exposure to mild intermittent hypoxia (16% O2, 4 h/day for 4 weeks) enhances spatial learning and memory in developing mice. However, whereas cell mechanisms involved remain poorly understood, no information was available on the long-term consequences on the aging brain of the surnumerary neurons produced in early life. In the present work, we first showed that within a few hours following hypoxia (evidenced by a significant PO_2_ decrease), specific brain areas such as the subventricular zone and the hippocampus achieved a reduced activation of pathways involved in cell death, such as those mediated by p38 MAP kinase and DAP kinase [34], [35]. By the same time, ERK1/2 pathway was upregulated, and this could be related to cell survival and cell proliferation reflected by increased BrdU incorporation, whereas the pro-survival Akt pathway may be not involved in our model. The presence of additional cells was confirmed at 21 days of age by enlarged size of various cell layers in the hippocampus, increased cell density, along with increased synaptic plasticity in line with higher levels of synapsins and phosphosynapsins. The importance of synapsin phosphorylation in the modulation of synapsin function and thus of neuronal activity has been largely documented. In this respect, phosphorylation on Ser^9^ residue has been reported to promote neurite outgrowth and to take part in the chain of molecular events that lead to the formation and stabilization of synapses, implying cell connections and enhanced neuronal survival [22]. In addition, the augmentation of glutamatergic and GABAergic cell phenotypes in various brain areas may not only reflect the conservation of a proper balance between the major excitatory and inhibitory systems, but may also constitute a hallmark of early hypoxia-related developmental events such as proliferation, migration, and differentiation [36].

Although the birth of newly generated cells appeared to be limited to the known germinative areas, i.e. the subventricular zone and the subgranular zone of the dentate gyrus, several lines of evidence suggest that new neurons may migrate widely from these neurogenic regions to be then broadly located in the brain [9], [32]. Thus, the question arises about the functional outcome of these surnumerary cells that potentially integrate existing circuitries. Secondarily, one may question whether these mechanisms trigger only temporary brain response or lead to prolonged brain adaptations, including during aging.

Aging is normally associated with functional decline of brain, especially cognitive decline which involves various mechanisms, including changes in synaptic transmission efficiency that play a central role in processes such as learning and memory or neurodegeneration. Although these mechanisms usually begin to occur earlier, during adulthood, behavioral parameters are the latest characteristics giving evidence of functional decline, and thus may be considered as alert signals of neurodegenerative processes [37]. In this respect, whereas the behavioral changes recorded are globally relatively modest, we report for the first time that male rats previously subjected to neonatal hypoxia displayed significantly higher scores than controls in a memory retrieval test during senescence, independently of their locomotor capacities. In fact, our data showed that these animals reached females’ scores, which were less affected by aging. Different studies using both cellular and functional markers have shown that, in normal conditions, female subjects present a higher resistance to senescence than males [38], [39]. Also, it has been documented that female rats have longer life expectancies compared to males and low rates of cerebral hemorrhage and vascular lesions until an advanced age [40]. Furthermore, obvious sex differences have been described in several brain regions involved in cognition such as the hippocampus [40], [41].

**Figure 10:**
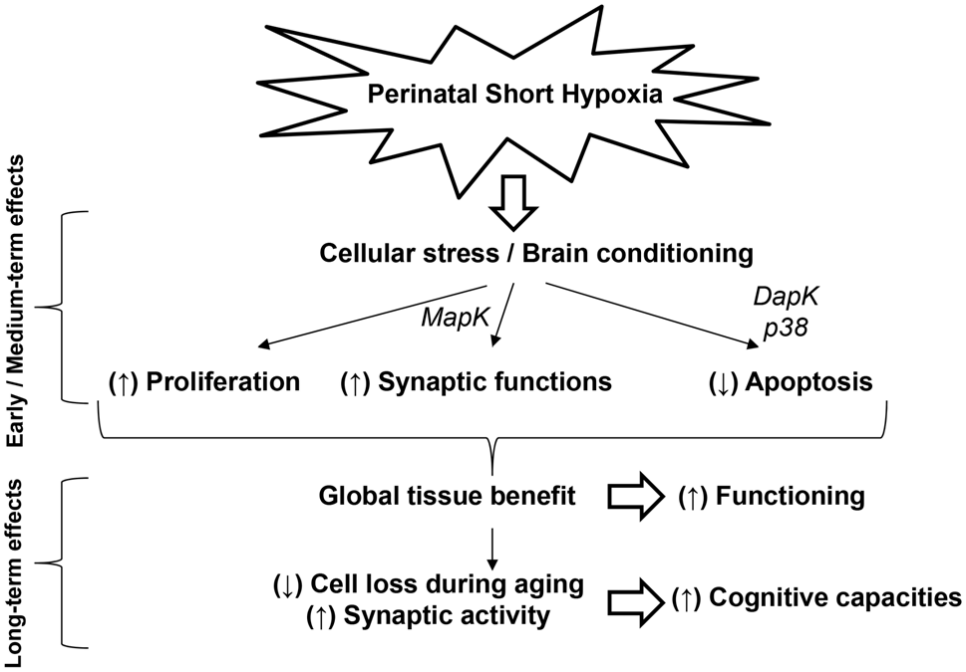
Summary of events involved in the hypoxia-mediated beneficial effects on the rat brain.

At the tissue level, our observation is consistent with the level of cytochrome oxidase activity in the dentate gyrus, a structure involved in memory. Actually, this measurement showed that, according to the existing correlation between global metabolic activity of the dentate gyrus and memory scores, aged males initially subjected to short hypoxia are closer to females than to paired control males. In addition, we showed that previously hypoxic 2-year-old males exhibited a better preservation of their brain integrity than controls, especially at the hippocampus level, with reduced age-related apoptosis, larger hippocampal cell layers, and higher expression of glutamatergic and GABAergic markers, as normally present at earlier ages and known to be involved in cognition. These changes were accompanied with a strong expression of synapsin proteins, mainly of their phosphorylated active forms which constitute key players of synapse function and plasticity [22], [42]. As shown in the present study, this latter observation does not only reflect the presence of a higher number of cells, but the activation of various, interdependent functional pathways involving Erk1/2 MAP kinases, the transcription factor EGR-1/Zif-268, and Src kinase with, ultimately, enhanced synapsin functions. The fact that the expression of these proteins remained unchanged in the brain of female rats would support the participation of the corresponding regulatory cascades in cognitive function. Our data are in good agreement with the demonstration that synaptic activity reduces apoptosis and promotes neuronal survival [43]. Furthermore, synaptic plasticity, such as LTP and LTD, exerts a central role in the acquisition and storage of information [44]. The NMDA receptors subserve synaptic glutamate-associated transmission and plasticity in central neurons, notably in the hippocampus. They are embedded in the postsynaptic density (PSD), a highly organized protein network where signal transduction at excitatory synapses along with information storage underlying learning and memory take place [45]. Studies on PSD-95 mutant mice have shown that PSD-95 is essential for mediating NMDA receptor-related LTD and LTP [29]. PSD-95 contributes to glutamatergic transmission and to the determination of the size and strength of synapses through a direct binding with NMDA receptors [46]. Accordingly, the significantly higher interactions between PSD-95 and NR2A receptor subunits measured in the hippocampus of 720-day-old male rats previously exposed to transient neonatal hypoxia strengthen the conclusion of increased synaptic functional activity and plasticity in those animals, in association with enhanced cognitive capacities. Taken together, our data show that an acute episode of hypoxia around birth can trigger neurogenesis associated with long lasting, beneficial rather than deleterious effects on the brain. The main findings are tentatively summarized in Figure 10. These observations are of particular interest with regard to the recent review by Simon N. Basovich [15] that questions about the capacity of early hypoxia to increase mental capacity and, as a possible consequence, its role in the etiology of mental disorders. The present results suggest that in some brain areas, such as the hippocampus, of aged males having experienced brief neonatal hypoxia, the quantity of neurons with increased synaptic functioning and plasticity could remain above a minimum threshold to confer higher resistance to senescence, with a better preservation of cognitive functions.

## Materials and Methods

### Ethics

Experiments were performed on Wistar rats (Charles River, l'Arbresle, France). They were conducted in accordance with internal guidelines (Nancy-University Ethic Comity) for animal care and housing, and meet all applicable standards for the ethics of experimentation and research integrity.

### Exposure to Hypoxia

Litters were reduced to 10 pups for homogeneity. Within 24 hours after birth, half of the litter was placed for 5 min in a thermoregulated Plexiglas chamber flushed with 100% N2, whereas the remaining pups were taken as controls and exposed for the same time to 21% O2/79% N2 (a mixture corresponding to air), as previously described [9]. The temperature inside the chamber was adjusted to 36°C to maintain body temperature in the physiological range. All pups were allowed to recover for 20 min in normoxic conditions, and were then returned to their dams. The hypoxic treatment did not result in any animal lethality. In some experiments, indices of hypoxia were assessed by the measurement of blood gases (PO2, PCO2, and pH), as previously described [47]. For this purpose, at the end of exposure to hypoxia (or to normoxia for controls), mixed blood samples were rapidly collected following decapitation, sheltered from ambient air, and rapidly injected into a gas analyzer (Corning Medical and Scientific, Halstead, UK).

### Histopathological Analyses

For further tissue investigations, a total of 32 animals were timely euthanized by decapitation (at 21 or 720 days of age). Brains were quickly harvested, frozen in methylbutane at −30°C, and kept at −80°C. Twelve-µm sagittal cryo-sections were generated, starting from the zero plane that bissects the brain mid-sagittally, and brain structures were identified according to the atlas of Sherwood and Timiras [48] or Paxinos and Watson [49]. For subsequent stainings and labeling counts, selected slides were coded prior to analysis and the codes were not broken until the experiments were completed.

Absolute numbers of labeled cells were estimated stereologically by means of the fractionator method, as described by Noori and Fornal [50], in 1 in 3 or 1 in 5 Dapi-stained 12-µm sagittal sections, depending on the brain region examined, the type of labeling, and the age of the individuals. For this purpose, a 100×100 µm grid was superimposed over each image-section and labeled cells were counted in 10 000 µm^2^ counting areas using a 20× objective. Total number of labeled cells (N) was calculated according to the equation: N = ΣQ−×(1/ssf )×(1/asf )×(1/tsf ), with Q− = number of counts, ssf = section sampling fraction, asf = area sampling fraction, and tsf = thickness of the sampling fraction. Calculation of the coefficient of error (CE(acf)) as an estimator of accuracy of the probe runs was performed according to the equation: CE = 0.039×acf –0.39, with acf = area of the counting frame [50]. In our investigations, CE(acf) = 0.001.

For basic histopathological investigations, brain sections were stained with thionin or the DNA fluorochrome 4,6-diamidino-2-phenylindole (Dapi, Sigma-Aldrich) for the measurement of layer thickness or global cell density by counting cell nuclei.

The presence of apoptosis was selectively depicted in tissue sections by the Apostain® method using monoclonal antibody to single-stranded DNA (F7-26, AbCys SA, Paris, France) after DNA denaturation by heating in the presence of formamide, as described by Blaise et al. [51].

### Labeling of Newly Generated Cells

To evaluate the rate of cell proliferation in response to hypoxia, bromodeoxyuridine (BrdU, Sigma-Aldrich, Saint-Quentin Fallavier, France) was solubilized in 0.9% NaCl containing 0.007 N NaOH, and administered intraperitoneally (50 mg/kg) as a single injection at various ages between 10 and 30 postnatal days. Animals were killed 24 h later by decapitation, and their brains immediately frozen in methylbutane at −30°C. For BrdU immunostaining, DNA was first denatured by incubating brain sections in 2 N HCl for 45 min at room temperature followed by a 10-min neutralization in 0.1 M sodium borate at pH 8.5. Tissue was rinsed in phosphate-buffered saline (PBS) for 10 min, then in PBS containing 10% goat serum for 1 h, and incubated overnight at 4°C with a mouse monoclonal antibody against BrdU (1/100, Oncogene Research Products, Boston, MA). After washing steps, brain sections were incubated in the presence of a Cy3-conjugated secondary antibody (1/100, Sigma-Aldrich) for subsequent detection of BrdU.

### Regional Brain Metabolism

Overall metabolic activity was evaluated in various brain areas by cytochrome oxidase (CO) histochemistry as originally reported by Wong-Riley [52] and slightly modified by Strazielle et al. [53]. Brain tissue sections were incubated in the dark for 75 min at 37°C in a continuously stirred solution of 0.1 M PBS (pH 7.4) containing 0.55 mg/mL 3,3'-diaminobenzidine tetrachloride (DAB, Sigma-Aldrich), 0.22 mg/mL horse-heart cytochrome c (Sigma-Aldrich), 0.2 mg/mL catalase (Sigma-Aldrich) and 44.5 mg/mL sucrose. Slides were washed in ice-cold buffer and immersed in a 10% buffered formalin solution for 30 min. They were then washed in buffer at room temperature, dehydrated in successive ethanol and xylene baths, and coverslipped with mounting medium. Brain sections from previously hypoxic and matched control rats were processed in parallel.

Enzyme activity was quantified by densitometric analysis by means of a computerized image-processing system (Biocom), and by using freshly prepared calibrated standards to convert absorbance into enzymatic activity as µmol/min per g of tissue.

### Tissue Immunochemistry

For immunohistochemical analyses, 12-µm brain sections were incubated in 0.1% triton X100 in PBS for 20 min at room temperature. Slides were dipped in PBS for 10 minutes, then in PBS containing 10% bovine serum for 1 h, and were incubated overnight at 4°C with a primary antibody against one of the following proteins: vesicular glutamate transporter 2 (VGlut2, mouse monoclonal, 1/100, Chemicon International, Millipore, Guyancourt, France), glutamate decarboxylase 65 (GAD65, rabbit polyclonal, 1/100, Chemicon International), Ox-6 (MHC Class II, mouse monoclonal,1/100, AbDserotec, Düsseldorf, Germany), Neurofilament 68 (NF68, rabbit polyclonal, 1/200, USBiological, Euromedex, Souffelweyersheim, France), Neuronal Nuclei protein (NeuN, mouse monoclonal, 1/200, Chemicon International), synapsin I (Syn1, rabbit polyclonal,1/200,Calbiochem, Merk, Lyon, France), synapsin II (Syn2, rabbit polyclonal, Abcam, Paris, France), phospho-synapsin (Pser9-synapsin, 1/200, rabbit polyclonal, Cell Signaling Technology, Ozyme, Saint Quentin en Yvelines, France), phospho-tyrosine (mouse monoclonal, 1/200, Cell Signaling Technology), phospho-serine (mouse monoclonal, 1/200, Cell Signaling Technology), NMDA NR2A (rabbit polyclonal, 1/200, Abcam), PSD-95 (mouse monoclonal, 1/200, Abcam), Ki67 (rabbit polyclonal, 1/200, Abcam).

After a washing step, immunoreactivity was depicted by incubation in the presence of an appropriate secondary anti-IgG antibody conjugated to AlexaFluor (1/1 000, Molecular Probes, Cergy-Pontoise, France). Immunofluorescence visualization was performed with a BX51WI microscope (Olympus, Rungis, France) and analyzed by Cell® analysis software (Olympus).

In addition, the “proximity ligation” assay (Duolink® *in situ* PLA™ reagents, Olink Bioscience, Eurogentec, Angers, France) was used to visualize and quantify synapsin coupling with phospho-Serine^9^ and phospho-Tyrosine^301^, as well as the interaction between NMDA NR2A subunits and PSD-95. A pair of oligonucleotide labeled secondary antibodies (PLA probes) generates a signal only when the two probes have bound in close proximity. The signal from each detected pair is visualized as an individual fluorescent dot. The PLA signals were counted based on microscopy images (Olympus BX51WI microscope with BlobFinder freeware from the Centre for Image Analysis, Uppsala University, Sweden).

### Western Blot Studies

Western blot analyses were performed on nitrogen frozen isolated brain structures. Tissue was solubilized in Ripa lysis buffer containing 140 mM NaCl, 0.5% (w/v) sodium deoxycholate, 1% (v/v) Nonidet P-40, 0.1% (w/v) SDS, and protease inhibitors (Complete, Roche Applied Science, Meylan, France). After homogenization, samples were lysed by three cycles of freezing/thawing and finally centrifuged at 4°C for 30 min at 15,000×*g*. The protein concentration in the supernatant was determined using the BCA protein assay kit (Pierce, Interchim, Monluçon, France). Forty µg of protein samples were mixed with an equal volume of 2× Laemmli buffer, denatured by heating the mixture for 5 min at 100°C, and then resolved by 12% SDS-PAGE. The separated proteins were transferred using a Mini Trans-Blot cell onto polyvinylidene fluoride membrane (Immobilon-P, Millipore), and the membranes were blocked for 1 h with Tris-buffered saline (pH 7.4) and 0.1% (v/v) Tween 20 (TBST buffer) containing 5% (w/v) bovine serum albumin. The polyvinylidene fluoride membrane were then incubated overnight at 4°C with a primary antibody against one of the following proteins: Akt (C67E7, rabbit monoclonal, 1/1000, Cell Signaling Technology, Beverly MA, USA), phospho-Akt (D9E, rabbit monoclonal, 1/1000, Cell Signaling Technology), p38 (rabbit polyclonal, 1/1000, Cell Signaling Technology), phospho-p38 (28B10, rabbit polyclonal, 1/1000, Cell Signaling Technology), Erk1/2 (rabbit polyclonal, 1/5000, Cell signaling Technology), phospho-Erk (Thr^202^/Tyr^204^, rabbit monoclonal, 1/500, Cell signaling Technology), death-associated protein kinase (clone DAPK-55, mouse monoclonal, 1/1000, Sigma-Aldrich), phospho-death-associated protein kinase (clone DKPS308, mouse monoclonal, 1/500, Sigma-Aldrich), EGR-1 (rabbit polyclonal, 1/500, Cell Signaling Technology), synapsin I (rabbit polyclonal, 1/2000, Calbiochem), synapsin II (rabbit polyclonal, 1/2000, Abcam), Src (36D10, rabbit monoclonal, 1/1000,Cell Signaling Technology), Glyceraldehyde-3-phosphate dehydrogenase (GAPDH, mouse monoclonal, 1/1,000, Abcam) was used as an internal standard. Polyvinylidene difluoride membranes were incubated for 1 h at room temperature with the corresponding horseradish peroxidase-conjugated preadsorbed secondary antibody (1/5000, Molecular Probes). Quantity One software, associated with the VersaDoc imaging system (Model 1000, Bio-Rad Laboratories), was used to quantify signals.

### Real-Time Quantitative RT-PCR

Total RNA was purified from nitrogen frozen brain tissue with the RNeasy Lipid Tissue kit following the manufacturer’s recommendations (Qiagen, Courtaboeuf, France), which included treatment with DNase. To evaluate possible DNA contamination of the RNA samples, reactions were also performed in control condition without Omniscript RT enzyme (Qiagen, Courtaboeuf, France).

Specific amplifications were performed using the following primers, forward: 5′-AGCTCCAGGATAAGGGAGGA-3′, and reverse: 5′-TGCCACGTGAAGAGCTGTC-3′ (expected product size: 140 bp) for death-associated protein kinase (DAPK); forward: 5′-GAGGCCCTCCACAACCAG-3′, and reverse: 5′-CTGCTGTGGGACTTGGTAGG-3′ (expected product size: 147 bp) for synapsin I, and forward: 5′-CATGGGTGTTTGCTCAGATG-3′, and reverse: 5′-TCTCTCGGTGATTGGGGTAG-3′ (expected product size: 96 bp) for synapsin II. Quantitation was performed using ribosomal protein S29 (RPS29) as an internal standard with the following primers: forward, 5′-ATGGGTCACCAGCAGCTCTA-3′ and reverse: 5′-GCCCGTATTTACGGATCAGA-3′ (expected product size: 106 bp). Real-time polymerase chain reaction was performed using the DNA binding dye SYBR Green I for the detection of polymerase chain reaction products. Temperature cycling for the DAPK run was 15 min at 95°C to activate the enzyme, followed by 50 cycles of 95°C for 10 seconds, 58°C for 15 seconds, and 72°C for 10 seconds. Temperature cycling for the synapsin I run was 15 min at 95°C to activate the enzyme, followed by 43 cycles of 95°C for 10 seconds, 57°C for 15 seconds, and 72°C for 10 seconds. Temperature cycling for the synapsin II run was 15 min at 95°C to activate the enzyme, followed by 43 cycles of 95°C for 10 seconds, 57°C for 15 seconds, and 70°C for 15 seconds. Temperature cycling for RPS29 run was 15 min at 95°C to activate the enzyme, followed by 43 cycles consisting of 95°C for 10 seconds, 57°C for 15 seconds, and 72°C for 10 seconds. Then melting curves analyses were performed by increasing temperature from 67 to 94°C. Calculation of the results was done with the RelQuant software (Roche Diagnostics, Manheim, Germany). Results were expressed as arbitrary units by calculating the ratio of crossing points of amplification curves of sample mRNAs and internal standard.

### Neurobehavioral Studies

Behavioral tests were performed between postnatal days 14 and 720 in order to evaluate the rats’ abilities and their cognitive performances as previously described [54], [55]. The data were analyzed in the light of gender, with a total of 16 controls (7 males and 9 females) and 15 previously exposed to hypoxia (9 males and 6 females).

#### Open-field test

To monitor psychomotor capacities, general activity was measured in an open-field from 180 to 720 days of age. At various time points, each rat performed a one-trial test consisting in behave freely in a circular area (90 cm in diameter and walls of 35 cm high). Four objects were suspended over the area in order to increase the interest of animals. These objects, corresponding to geometric forms, were changed in size and colour between trials. Their height position was chosen to be easily seen by rats without any contact possible. The open-field was carefully washed between every assay. The total time for each test was fixed at 3 min and two parameters were collected: the time spent by rats in locomotor activity (corresponding to the total time of the test minus the total time of immobility) and the total amount of rearing behaviours (counted each time the rat leaved the ground with his front paws).

#### Multiple T-maze

Animals were tested for learning and memory (reference memory) in a multiple T-maze with 6-choice points and dimensions of 180×110 cm. The alley through which the animals navigated had a height of 35 cm and a width of 8 cm. It is assumed that food deprivation motivates animals to reach the goal box where they would be rewarded with food. Rats deprived of food for 24 h were trained two times per day for five consecutive days between postnatal days 14 and 18. Memory retrieval for this maze was tested at various ages, from 180 to 720 days, by one single run. Time to reach the goal as well as wrong decisions at the choice points (number of errors) were recorded. For homogeneity, tests were always performed between 8∶00 and 11∶00 a.m.

### Statistical Analyses

Data were prospectively collected and analyzed with Statview 5 software for Windows (SAS Institute, Berkley, CA). Raw data were compared by using one-way analysis of variance (ANOVA) with Fisher’s test. For all analyses, a *P* value<0.05 was considered to indicate statistical significance.

## Supporting Information

Figure S1 Lack of significant effects of neonatal hypoxia on brain histological characteristics and synapsin-related proteins in 720-day-old female rats. (**A**) Layer thicknesses in the hippocampus of control and hypoxic female rats (n = 5, DG = dentate gyrus). (**B**) Number of apoptotic cells as depicted by the Apostain® method in various brain areas (n = 5, PFCx = prefrontal cortex, FCx = frontal cortex,). (**C**) Expression of synapsin I (green) and NeuN (red) in the hippocampus (nuclei were counterstained by Dapi). (**D**) Western blot analysis of regulatory proteins of synapsins in the hippocampus of control (C) and hypoxic (H) rats and corresponding densitometric analyses. Data are expressed in arbitrary units (AU) as means ± SD (n = 3). Similar observations were made in the cortex (not shown).
(TIF)

Figure S2 Proliferating cells in the brain of aged male rats. Representative illustration of Ki67 labeling (red) in 720-day-old male rats in the two major germinative zones, the subventricular zone (SVZ) and the dentate gyrus (DG). Cells were counterstained by Dapi (blue). See results section for quantitative data.
(TIF)

## References

[1] Barker DJ (1992) The effect of nutrition of the fetus and neonate on cardiovascular disease in adult life. Proc Nutr Soc 51: 135–144. 1438321 10.1079/pns19920023

[2] McEwen BS (2003) Early life influences on life-long patterns of behavior and health. Ment Retard Dev Disabil Res Rev 9: 149–154. 12953293 10.1002/mrdd.10074

[3] Weaver IC (2009) Shaping adult phenotypes through early life environments. Birth Defects Res 87: 314–326. 10.1002/bdrc.20164 19960543

[4] Lemaire V, Lamarque S, Moal ML, Piazza PV, Abrous DN (2006) Postnatal stimulation of the pups counteracts prenatal stress-induced deficits in hippocampal neurogenesis. Biol Psychiatry 59: 786–792. 16460692 10.1016/j.biopsych.2005.11.009

[5] Gidday JM (2006) Cerebral preconditioning and ischaemic tolerance. Nat Rev Neurosci 7: 437–448. 16715053 10.1038/nrn1927

[6] Bossenmeyer-Pourié C, Daval JL (1998) Prevention from hypoxia-induced apoptosis by preconditioning: a mechanistic approach in cultured neurons from fetal rat forebrain. Mol Brain Res 58: 237–239. 9685661 10.1016/s0169-328x(98)00123-5

[7] Bossenmeyer-Pourié C, Chihab R, Schroeder H, Daval JL (1999) Transient hypoxia may lead to neuronal proliferation in the developing mammalian brain, from apoptosis to cell cycle completion. Neuroscience 91: 221–231. 10336073 10.1016/s0306-4522(98)00565-x

[8] Ong J, Plane JM, Parent JM, Silverstein FS (2005) Hypoxic-ischemic injury stimulates subventricular zone proliferation and neurogenesis in the neonatal rat. Pediatr Res 58: 600–606. 16148080 10.1203/01.PDR.0000179381.86809.02

[9] Pourié G, Blaise S, Trabalon M, Nédélec E, Guéant JL, et al (2006) Mild, non-lesioning transient hypoxia in the newborn rat induces delayed brain neurogenesis associated with improved memory scores. Neuroscience 140: 1369–1379. 16650606 10.1016/j.neuroscience.2006.02.083

[10] Hastings NB (2001) Tanapat P, Gould E. Neurogenesis in the adult mammalian brain. Clin Neurosci Res 1: 175–182.

[11] Lie DC, Song H, Colamarino SA, Ming GL, Gage FH (2004) Neurogenesis in the adult brain, new strategies for central nervous system diseases. Annu Rev Pharmacol Toxicol 44: 399–421. 14744252 10.1146/annurev.pharmtox.44.101802.121631

[12] Martin N, Pourié G, Bossenmeyer-Pourié C, Jazi R, Guéant JL, et al (2010) Conditioning-like brief neonatal hypoxia improves cognitive function and brain tissue properties with marked gender dimorphism in adult rats. Semin Perinatol 34: 193–200. 20494735 10.1053/j.semperi.2010.02.003

[13] Zornberg GL, Buka SL, Tsuang MT (2000) Hypoxic-ischemia-related fetal/neonatal complications and risk of schizophrenia and other nonaffective psychoses, a 19-year longitudinal study. Am J Psychiatry 157: 196–202. 10671387 10.1176/appi.ajp.157.2.196

[14] Dalman C, Thomas HV, David AS, Gentz J, Lewis G, et al (2001) Signs of asphyxia at birth and risk of schizophrenia. Population-based case-control study. Br J Psychiatry 179: 403–408. 11689395 10.1192/bjp.179.5.403

[15] Basovich SN (2010) The role of hypoxia in mental development and in the treatment of mental disorders, a review. Biosci Trends 4: 288–296. 21248426

[16] Wullimann MF (2009) Secondary neurogenesis and telencephalic organization in zebrafish and mice, a brief review. Integr Zool 4: 123–133. 21392282 10.1111/j.1749-4877.2008.00140.x

[17] Failor S, Nguyen V, Darcy DP, Cang J, Wendland MF, et al (2010) Neonatal cerebral hypoxia-ischemia impairs plasticity in rat visual cortex. J Neurosci 30: 81–92. 20053890 10.1523/JNEUROSCI.5656-08.2010 PMC2822440

[18] Cuzon Carlson VC, Seabold GK, Helms CM, Garg N, Odagiri M, et al (2011) Synaptic and morphological neuroadaptations in the putamen associated with aong-term, relapsing alcohol drinking in primates. Neuropsychopharmacology 36: 2513–2528. 21796110 10.1038/npp.2011.140 PMC3194078

[19] Pamenter ME, Hogg DW, Ormond J, Shin DS, Woodin MA, et al (2011) Endogenous GABAA and GABAB receptor-mediated electrical suppression is critical to neuronal anoxia tolerance. Proc Natl Acad Sci USA 108: 11274–11279. 21690381 10.1073/pnas.1102429108 PMC3131309

[20] Lang JT, McCullough LD (2008) Pathways to ischemic neuronal cell death, are sex differences relevant? J Transl Med 6: 33. 18573200 10.1186/1479-5876-6-33 PMC2459157

[21] Renolleau S, Fau S, Charriaut-Marlangue C (2008) Gender-related differences in apoptotic pathways after neonatal cerebral ischemia. Neuroscientist 14: 46–52. 17971506 10.1177/1073858407308889

[22] Cesca F, Baldelli P, Valtorta F, Benfenati F (2010) The synapsins, key actors of synapse function and plasticity. Prog Neurobiol 91: 313–348. 20438797 10.1016/j.pneurobio.2010.04.006

[23] Vara H, Onofri F, Benfenati F, Sassoè-Pognetto M, Giustetto M (2009) ERK activation in axonal varicosities modulates presynaptic plasticity in the CA3 region of the hippocampus through synapsin I. Proc Natl Acad Sci USA. 106: 9872–9877. 10.1073/pnas.0900077106 PMC2701005 19487674

[24] Giachello CN, Fiumara F, Giacomini C, Corradi A, Milanese C, et al (2010) MAPK/Erk-dependent phosphorylation of synapsin mediates formation of functional synapses and short-term homosynaptic plasticity. J Cell Sci 123: 881–893. 20159961 10.1242/jcs.056846

[25] Thiel G, Schoch S, Petersohn D (1994) Regulation of synapsin I gene expression by the zinc finger transcription factor zif268/egr-1. J Biol Chem 269: 15294–15301. 8195167

[26] Petersohn D, Schoch S, Brinkmann DR, Thiel G (1995) The human synapsin II gene promoter. Possible role for the transcription factor zif268/egr-1, polyoma enhancer activator 3, and AP2. J Biol Chem 270: 24361–24369. 7592648 10.1074/jbc.270.41.24361

[27] Kaufmann K, Bach K, Thiel G (2001) The extracellular signal-regulated protein kinases Erk1/Erk2 stimulate expression and biological activity of the transcriptional regulator Egr-1. Biol Chem 382: 1077–1081. 11530939 10.1515/BC.2001.135

[28] Messa M, Congia S, Defranchi E, Valtorta F, Fassio A, et al (2010) Tyrosine phosphorylation of synapsin I by Src regulates synaptic-vesicle trafficking. J Cell Sci 123: 2256–2265. 20530578 10.1242/jcs.068445

[29] Xu W (2011) PSD-95-like membrane associated guanylate kinases (PSD-MAGUKs) and synaptic plasticity. Curr Opin Neurobiol 21: 306–312. 21450454 10.1016/j.conb.2011.03.001 PMC3138136

[30] Cousins SL, Stephenson FA (2012) Identification of N-methyl-D-aspartic acid (NMDA) receptor subtype-specific binding sites that mediate direct interactions with scaffold protein PSD-95. J Biol Chem 287: 13465–13476. 22375001 10.1074/jbc.M111.292862 PMC3339998

[31] Taupin P (2006) Adult neurogenesis in mammals. Curr Opin Mol Ther 8: 345–351. 16955698

[32] Ohira K (2011) Injury-induced neurogenesis in the mammalian forebrain. Cell Mol Life Sci 68: 1645–1656. 21042833 10.1007/s00018-010-0552-y PMC11115059

[33] Lu XJ, Chen XQ, Weng J, Zhang HY, Pak DT, et al (2009) Hippocampal spine-associated Rap-specific GTPase-activating protein induces enhancement of learning and memory in postnatally hypoxia-exposed mice. Neuroscience 162: 404–414. 19442707 10.1016/j.neuroscience.2009.05.011 PMC3243647

[34] Xia Z, Dickens M, Raingeaud J, Davis RJ, Greenberg ME (1995) Opposing effects of ERK and JNK-p38 MAP kinases on apoptosis. Science 270: 1326–1331. 7481820 10.1126/science.270.5240.1326

[35] Bialik S, Kimchi A (2006) The death-associated protein kinases, structure, function, and beyond. Annu Rev Biochem 75: 189–210. 16756490 10.1146/annurev.biochem.75.103004.142615

[36] Luján R, Shigemoto R, López-Bendito G (2005) Glutamate and GABA receptor signalling in the developing brain. Neuroscience 130: 567–580. 15590141 10.1016/j.neuroscience.2004.09.042

[37] Taffe MA, Weed MR, Gutierrez T, Davis SA, Gold LH (2004) Modeling a task that is sensitive to dementia of the Alzheimer's type, individual differences in acquisition of a visuo-spatial paired-associate learning task in rhesus monkeys. Behav Brain Res 149: 123–133. 15129776 10.1016/s0166-4328(03)00214-6

[38] Wiederholt WC, Cahn D, Butters NM, Salmon DP, Kritz-Silverstein D, et al (1993) Effects of age, gender and education on selected neuropsychological tests in an elderly community cohort. J Am Geriatr Soc 41: 639–647. 8505462 10.1111/j.1532-5415.1993.tb06738.x

[39] Coffey CE, Lucke JF, Saxton JA, Ratcliff G, Unitas LJ, et al (1998) Sex differences in brain aging, a quantitative magnetic resonance imaging study. Arch Neurol 55: 169–179. 9482358 10.1001/archneur.55.2.169

[40] Cahill L (2006) Why sex matters for neuroscience. Nat Rev Neurosci 7: 477–484. 16688123 10.1038/nrn1909

[41] Juraska JM (1991) Sex differences in ‘cognitive’ regions of the rat brain. Psychoneuroendocrinology 16: 105–109. 1961834 10.1016/0306-4530(91)90073-3

[42] Turner KM, Burgoyne RD, Morgan A (1999) Protein phosphorylation and the regulation of synaptic membrane traffic. Trends Neurosci 22: 459–464. 10481193 10.1016/s0166-2236(99)01436-8

[43] Léveillé F, Papadia S, Fricker M, Bell KF, Soriano FX, et al (2010) Suppression of the intrinsic apoptosis pathway by synaptic activity. J Neurosci 30: 2623–2635. 20164347 10.1523/JNEUROSCI.5115-09.2010 PMC2834927

[44] Silva AJ (2003) Molecular and cellular cognitive studies of the role of synaptic plasticity in memory. J Neurobiol 54: 224–237. 12486706 10.1002/neu.10169

[45] Garner CC, Nash J, Huganir RL (2000) PDZ domains in synapse assembly and signalling. Trends Cell Biol 10: 274–280. 10856930 10.1016/s0962-8924(00)01783-9

[46] Kim E, Sheng M (2004) PDZ domain proteins of synapses. Nat Rev Neurosci 5: 771–781. 15378037 10.1038/nrn1517

[47] Grojean S, Schroeder H, Pourié G, Charriaut-Marlangue C, Koziel V, et al (2003) Histopathological alterations and functional brain deficits after transient hypoxia in the newborn rat pup, a long term follow-up. Neurobiol Dis 14: 265–278. 14572448 10.1016/s0969-9961(03)00082-2

[48] Sherwood NM, Timiras PS (1970) A stereotaxic atlas of the developing rat brain. University of California Press, Berkeley.

[49] Paxinos G, Watson C (2007) The Rat Brain in Stereotaxic Coordinates (ed 6). Elsevier/Academic Press, New York.

[50] Noori HR, Fornal CA (2011) The appropriateness of unbiased optical fractionators to assess cell proliferation in the adult hippocampus. Front Neurosci 5: 140. 22207833 10.3389/fnins.2011.00140 PMC3245968

[51] Blaise SA, Nédélec E, Schroeder H, Alberto JM, Bossenmeyer-Pourié C, et al (2007) Gestational vitamin B deficiency leads to homocysteine-associated brain apoptosis and alters neurobehavioral development in rats. Am J Pathol 170: 667–679. 17255334 10.2353/ajpath.2007.060339 PMC1851855

[52] Wong-Riley MTT (1979) Changes in the visual system of monocularly sutured or enucleated cats demonstrable with cytochrome oxidase histochemistry. Brain Res 171: 11–28. 223730 10.1016/0006-8993(79)90728-5

[53] Strazielle C, Hayzoun K, Derer M, Mariani J, Lalonde R (2006) Regional brain variations of cytochrome oxidase activity in Relnrl-orl mutant mice. J Neurosci Res 83: 821–831. 16511878 10.1002/jnr.20772

[54] Hoeger H, Engelmann M, Bernert G, Seidl R, Bubna-Littitz H, et al (2000) Long term neurological and behavioral effects of graded perinatal asphyxia in the rat. Life Sci 66: 947–962. 10714895 10.1016/s0024-3205(99)00678-5

[55] Hoeger H, Engidawork E, Stolzlechner D, Bubna-Littitz H, Lubec B (2006) Long-term effect of moderate and profound hypothermia on morphology, neurological, cognitive and behavioural functions in a rat model of perinatal asphyxia. Amino Acids 31: 385–396. 16944046 10.1007/s00726-006-0393-z

