# Supplementary figures and images for: Non-Injurious Neonatal Hypoxia Confers Resistance to Brain Senescence in Aged Male Rats
Source: PLoS One. 2012 Nov 16;7(11):e48828. doi: 10.1371/journal.pone.0048828 (PMC3500249; doi:10.1371/journal.pone.0048828)

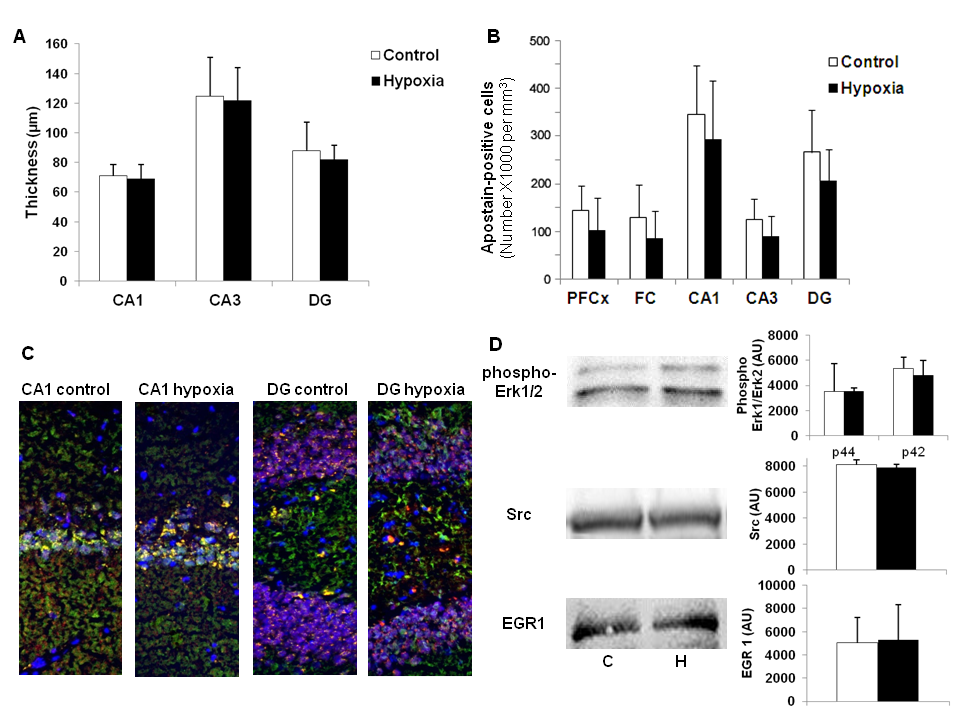

Supplement: Figure S1 — Lack of significant effects of neonatal hypoxia on brain histological characteristics and synapsin-related proteins in 720-day-old female rats. (A) Layer thicknesses in the hippocampus of control and hypoxic female rats (n = 5, DG = dentate gyrus). (B) Number of apoptotic cells as depicted by the Apostain® method in various brain areas (n = 5, PFCx = prefrontal cortex, FCx = frontal cortex,). (C) Expression of synapsin I (green) and NeuN (red) in the hippocampus (nuclei were counterstained by Dapi). (D) Western blot analysis of regulatory proteins of synapsins in the hippocampus of control (C) and hypoxic (H) rats and corresponding densitometric analyses. Data are expressed in arbitrary units (AU) as means ± SD (n = 3). Similar observations were made in the cortex (not shown). (TIF) [file pone.0048828.s001.tif]

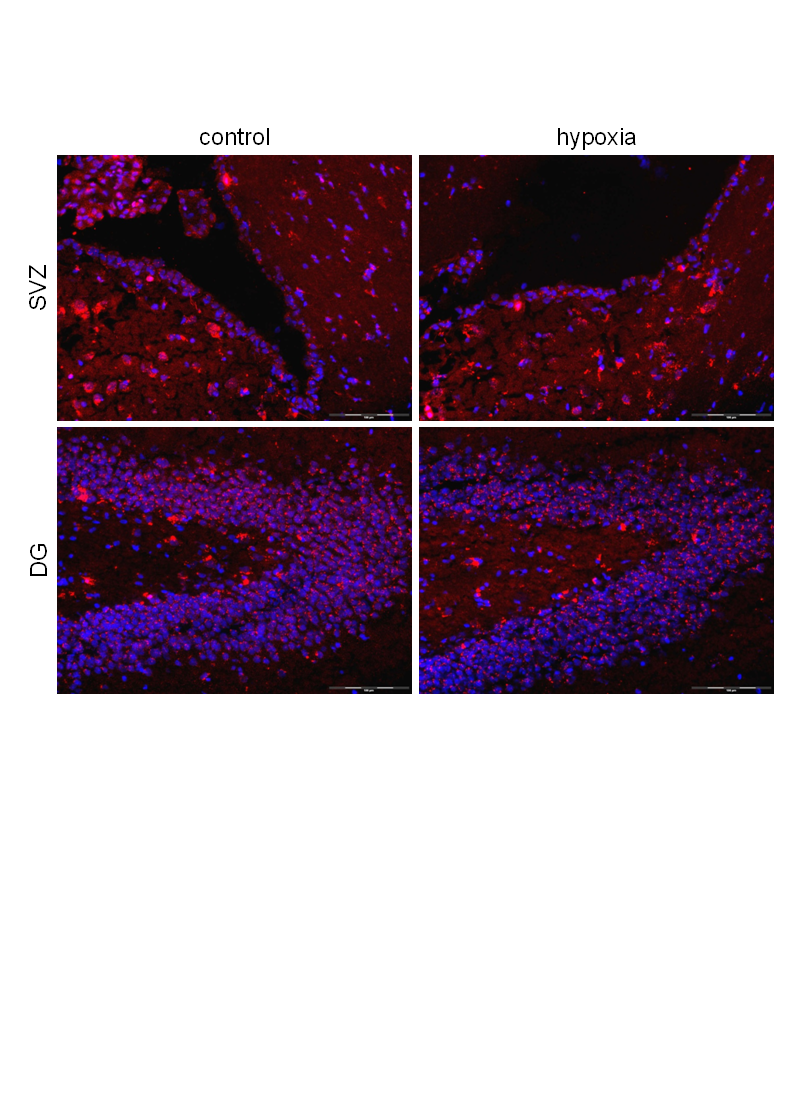

Supplement: Figure S2 — Proliferating cells in the brain of aged male rats. Representative illustration of Ki67 labeling (red) in 720-day-old male rats in the two major germinative zones, the subventricular zone (SVZ) and the dentate gyrus (DG). Cells were counterstained by Dapi (blue). See results section for quantitative data. (TIF) [file pone.0048828.s002.tif]
